# Bacterial and Archaeal Diversity in an Iron-Rich Coastal Hydrothermal Field in Yamagawa, Kagoshima, Japan

**DOI:** 10.1264/jsme2.ME13048

**Published:** 2013-11-21

**Authors:** Satoshi Kawaichi, Norihiro Ito, Takashi Yoshida, Yoshihiko Sako

**Affiliations:** 1 Laboratory of Marine Microbiology, Graduate School of Agriculture, Kyoto University, Kyoto 606–8502, Japan

**Keywords:** coastal hydrothermal field, thermophiles, chemo-synthetic ecosystem, extremophiles

## Abstract

Physicochemical characteristics and archaeal and bacterial community structures in an iron-rich coastal hydrothermal field, where the temperature of the most active hot spot reaches above 100°C, were investigated to obtain fundamental information on microbes inhabiting a coastal hydrothermal field. The environmental settings of the coastal hydrothermal field were similar in some degree to those of deep-sea hydrothermal environments because of its emission of H_2_, CO_2_, and sulfide from the bottom of the hot spot. The results of clone analyses based on the 16S rRNA gene led us to speculate the presence of a chemo-synthetic microbial ecosystem, where chemolithoautotrophic thermophiles, primarily the bacterial order *Aquificales*, function as primary producers using H_2_ or sulfur compounds as their energy source and CO_2_ as their carbon source, and the organic compounds synthesized by them support the growth of chemoheterotrophic thermophiles, such as members of the order *Thermales* and the family *Desulfurococcaceae*. In addition, the dominance of members of the bacterial genus *Herbaspirillum* in the high temperature bottom layer led us to speculate the temporal formation of mesophilic zones where they can also function as primary producing or nitrogen-fixing bacteria.

Hydrothermal systems, where geothermally heated waters are expelled through fissures in the Earth’s crust, are located both on land and under the sea. Deep-sea hydrothermal systems are one of the most extensively studied habitats of thermophilic microorganisms, since the first deep-sea hydrothermal vent on the Galapagos Rift and its associated macrofauna were discovered in 1977 (10). In deep-sea hydrothermal systems, where no sunlight penetrates, microbial primary production is achieved by chemolithoautotrophs utilizing H_2_, sulfur-bearing compounds, and CO_2_ (3, 6, 20, 21, 27, 36, 55). These compounds are provided by magma degassing and reactions between seawater and rock at high temperatures (6, 66), and serve as energy (electron donor/acceptor) and carbon sources.

Compared to deep-sea hydrothermal systems, considerably less is known about the microbial community structures inhabiting shallow-sea systems, such as on-shore seeps and shallow vents. Like their deep-sea counterparts, shallow-sea hydrothermal systems are often found in volcanic regions (*e.g.* New Zealand, Italy, and Argentina). For example, the presence of shallow vents located in a few meters of water on Vulcano Island, Italy have been known for much longer than deep-sea systems due to their proximity to the shore and easier accessibility and also some microbes have been isolated from the vents (2, 51, 56). These shallow-sea systems differ from deep-sea systems in a variety of ways. For example, in shallow-sea systems, hydrothermal fluids are much cooler than deep-sea systems where fluids can surpass 400°C due to high hydrostatic pressure (4). The chemical composition of hydrothermal fluids is reducing, like deep-sea or terrestrial hydrothermal sites, but shallow-sea system fluids are usually richer in N, P, and Si and do not have such high concentrations of CH_4_ and H_2_ as deep-sea systems (66). Shallow-sea vent fluids often contain a meteoric water component (47) and their chemistry can affect near-shore activity and vice versa (46). These unique environmental conditions led us to expect that the microbial inhabitants of shallow-sea hydrothermal fields could be distinct from those of other environments, including deep-sea hydrothermal systems. However, microbial communities in shallow-sea hydrothermal systems have so far garnered less interest than those in their deep-sea counterparts.

The Yamagawa coastal hydrothermal field is located in the Kirishima Volcanic Belt, which is one of the most active volcanic areas in Japan. Recently, two novel thermophilic bacteria were isolated from this environment (26, 61). In addition, strains of strictly aerobic, neutrophilic, hyperthermophilic archaeal genus *Aeropyrum* (Tanaka *et al.* unpublished data) and two novel strains of virus infecting the type species of the genus *Aeropyrum* were successfully isolated (38, 39). However, no study on the comprehensive microbial community has performed in this environment.

We first determined the fundamental physicochemical characteristics, such as the temperature, pore water characteristics, gas compositions, and the iron content of the sediments in the Yamagawa coastal hydrothermal field. The factors were inferred to have a critical effect on the spectrum of viable microbes in the environment. The compositions of pore water and gas gave information on the availability of electron donors/acceptors and carbon sources in the environment for microbes. Thereafter, we performed culture-independent analysis targeting the environmental 16S rRNA gene (16S rRNA gene clone analysis) to obtain a snap-shot of the microbial communities inhabiting the Yamagawa coastal hydrothermal field.

## Materials and Methods

### Study site description and sample collection

**Fig. 1:**
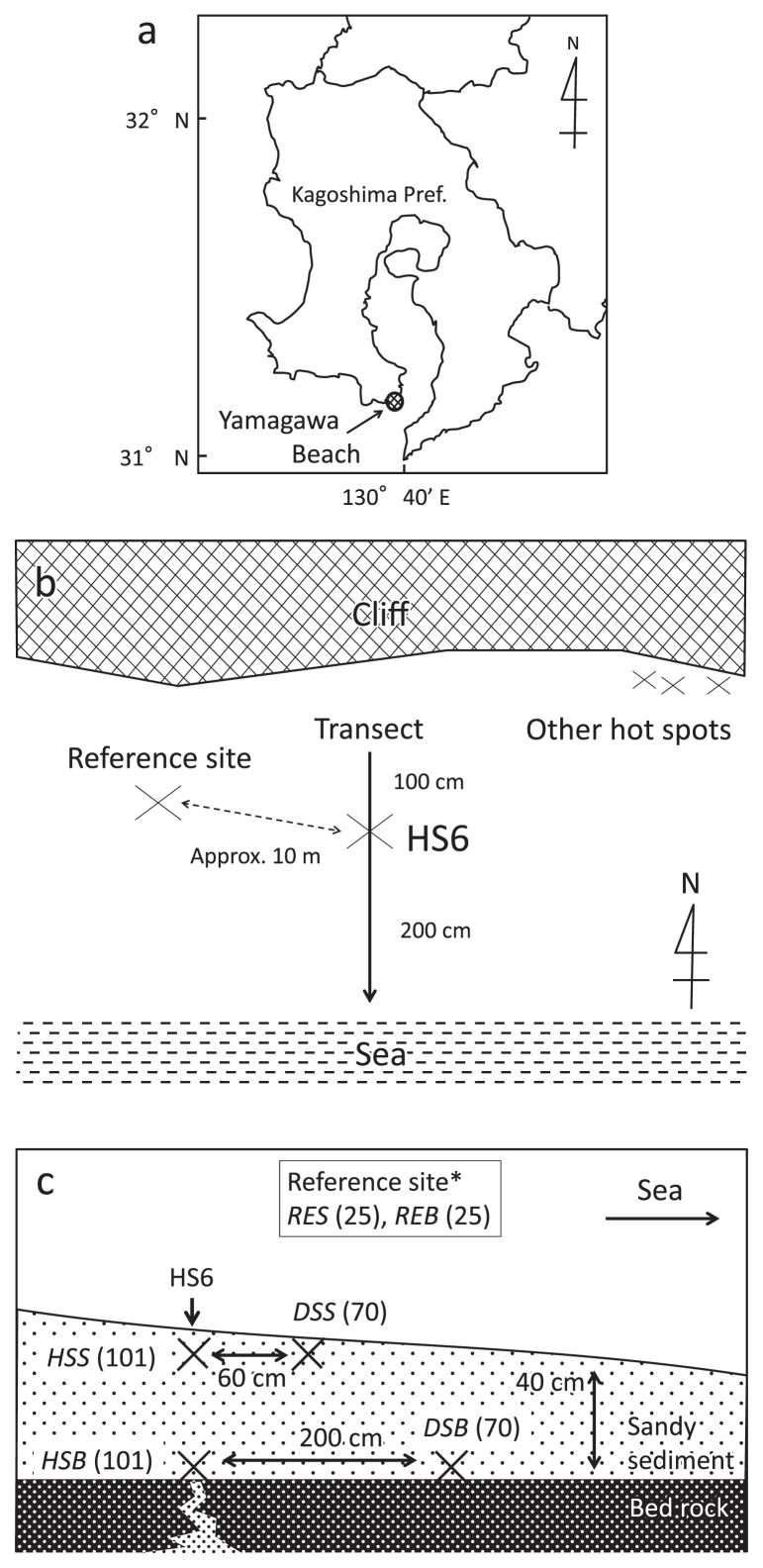
Location of Yamagawa Beach (a), transect across HS6 (b), and sampling sites in the Yamagawa coastal hydrothermal field (c). Sampling sites are shown in italic. See text for abbreviations. Numbers in parentheses indicate the temperature of the samples. *Reference site is off the transect, approximately 10 m from HS6.

The study site was a coastal hydrothermal field on the Yamagawa Beach (31°10′58″ N, 130°36′59″ E) (Fig. 1a), a coarse-grained sandy beach on the southern coast of Kyushu Island (Japan). Several hot spots erupt hot steam on the beach. The most active steam vent (Hot spot No. 6: HS6) was selected and a sampling transect was established through HS6 from the land to the sea (Fig. 1b). Sediments were collected from six points along the transect with different depths and temperatures (Fig. 1c) to determine the amount of ferric iron (Fe[III]) in the sediments, and for clone analyses. Each sample name is abbreviated to three letters: the first two letters for the temperature zones: HS for hot spot (101°C), DS for downstream (70°C), and RE for reference site (25°C), and the last letter for depth; S for surface (0–5 cm beneath surface), and B for bottom (approx. 40 cm beneath surface). Since we could not obtain vertical core samples due to the coarse-grained sandy and friable sediments, we dug a hole near each sampling point and collected the sediments using tip-cut syringes. Approximately 40 g (wet weight) of sediment was immediately transferred into a 50 mL glass vial and flushed with 100% N_2_ (100 kPa). The vial was closed tightly using a butyl rubber stopper and sealed with a screw cap. No reducing agent was added. Samples were stored at 4°C in the dark until used.

### Physicochemical measurements

The vertical temperature profile was determined *in situ* along the transect using a probe thermometer. Dissolved oxygen (DO), dissolved hydrogen sulfide, and pH of pore water were determined *in situ* using a CHEMets Kit for Dissolved Oxygen (K-7512 for 1–12 ppm, and K-7501 for 0–1.0 ppm; CHEMetrics, Midland, VA, USA), a twin pH Compact pH Meter (Horiba, Kyoto, Japan), and a VISOCOLOR ECO Sulphide kit (Marchery-Nagel, Düren, Germany), respectively. We could not determine DO, dissolved hydrogen sulfide, or pH of the pore water of the surface layer due to an insufficient amount of pore water.

Gas samples were collected from the bottom layer of each point (a sample was additionally collected from the surface layer of HS6) using a 50 mL syringe equipped with a 30 cm length needle. Gas contents were analyzed using gas chromatography (GC-2014; Shimadzu, Kyoto, Japan) equipped with a thermal conductivity detector and a SHINCARBON ST packed column (Shinwa Chemical Industries, Kyoto, Japan). Argon was used as the carrier gas.

Iron contents of the sediments were determined using the method described by Lovley and Phillips (33). This method is based on the reduction of poorly crystalline ferric iron (amorphous Fe[III] oxyhydroxide) by hydroxylamine under acidic conditions and colorimetry using ferrozine (59) as an indicator. Fe(III) exists in a variety of chemical forms. Among them, amorphous Fe(III) oxyhydroxide is known as one of the most preferable form for microbial Fe(III) reduction (32, 40). Hydroxylamine extraction is more selective for amorphous Fe(III) oxyhydroxide than acid ammonium oxalate extraction (33).

### DNA extraction and PCR amplification of bacterial and archaeal 16S rRNA genes

Genomic DNA for clone libraries was extracted from approximately 10 g of each sediment sample using an UltraClean Mega Soil DNA Isolation Kit (MO BIO Laboratories, Carlsbad, CA, USA) and the obtained DNA (8 mL) was concentrated to a volume of 500 μL with distilled water by following the manufacturer’s instructions. Bacterial and archaeal 16S rRNA genes were amplified by PCR using primers Bac27F (30) and Bac907R (41) for bacterial rRNA genes and primers Arch21F and Arch958R (11) for archaeal rRNA genes. Resultant PCR products were purified using an MinElute Gel Extraction Kit (Qiagen, Venlo, Netherlands). The purified DNA was cloned in the vector pCR2.1 using the Original TA cloning kit (Invitrogen, Carlsbad, CA, USA). The inserts were amplified by direct PCR from a single colony using a primer set of M13 primer M4 (5′-GTT TTC CCA GTC ACG AC-3′) and M13 primer RV (5′-CAG GAA ACA GCT ATG AC-3′), treated with exonuclease I and shrimp alkaline phosphatase (Amersham Pharmacia Biotech, Uppsala, Sweden), and directly sequenced. Primers Bac27F and Arch21F were used in partial sequencing analysis for *Bacteria* and for *Archaea*, respectively. Sequences shorter than 300 bp were omitted from further analyses.

### Sequence analysis

The rRNA gene sequences with ≥95% similarity were assigned to the same clone type (Operational Taxonomic Unit, OTU) in both bacterial and archaeal libraries using the FastGroupII program (69). Also, a representative rRNA gene clone of each OTU was determined and rarefaction curves were obtained for each clone library using the same program. Each representative sequence was subjected to an initial pre-classification step using the RDP classifier (67), in which unclassified sequences (not *Archaea* or *Bacteria*) or sequences classified into the incorrect domain (*e.g. Bacteria* in the archaeal data set) were removed. Each OTU was assigned to a phylum (for *Bacteria*, instead of proteobacterial OTUs, which were assigned to a class) or to a family (for *Archaea*) using the RDP classifier (67) set at a bootstrap value of 80% (9). Manual classification using maximum-likelihood (ML) trees (described below) and the Blastn search were also performed for unclassified OTUs, since some known lineages (*e.g. Thaumarchaeota*) were not classified by the RDP classifier.

### Phylogenetic analyses

The obtained representative sequences of each OTU were subjected to a BLASTN search (NCBI: http//www.ncbi.nlm.nih.gov/). The best hit sequence for each representative sequence was collected and used along with the sequences obtained in this study for the following phylogenetic analyses. In order to determine the phylogenetic positions of the representative sequences, sequences were aligned using the ClustalW program (31) available online at the DDBJ (DNA Data Bank of Japan) web site. The ML trees were inferred by the PhyML program in the Phylogeny.fr. platform (12, 19).

### Comparison of clone libraries

Differences in bacterial and archaeal 16S rRNA gene libraries (beta-diversity) were assessed using phylogeny-based metrics, UniFrac (34). Sequences were subjected to the UniFrac program with their “environment file,” which describes the source sample of each sequence and the ML tree obtained as described above. UniFrac distances were calculated to account for the abundance of individual taxa (weighted-UniFrac PCA [principal coordinates analysis]). While the libraries consist of different numbers of sequences, UniFrac PCA with a normalized algorithm was performed.

The sequences reported in this study were deposited in the DDBJ database under DDBJ/EMBL/GenBank accession numbers AB812106–AB812548.

### Quantitative analyses of bacterial and archaeal 16S rRNA genes

The bacterial and archaeal 16S rRNA genes in each sample were quantified by a quantitative fluorescent PCR method using the Thermal Cycler Dice Real Time System Single (TaKaRa Bio, Otsu, Japan), and SYBR Premix Ex Taq (TaKaRa Bio) and SYBR Premix Ex Taq II (TaKaRa Bio) were used for quantitative detection of the bacterial 16S rRNA gene and archaeal 16S rRNA gene, respectively, following the manufacturer’s protocol as previously described (68), while the reaction volume was reduced to 12.5 μL. For bacterial and archaeal 16S rRNA genes, respectively, primer sets 338f-518r and 931f-m1100r were used as described previously (15). For external standards, we used genomic DNA of *Escherichia coli* DH5α for the bacterial 16S rRNA gene, and *Aeropyrum pernix* K1^T^ (= JCM 9820^T^) for the archaeal 16S rRNA gene.

## Results and Discussion

### Physicochemical characteristics

The maximum temperature of the sediment in the bottom layer (approximately 30 cm beneath sediment surface) of HS6 was 104°C with an average temperature of 101°C (Table 1). The temperature of the sediment decreased in proportion to the distance from HS6. A steep gradient of temperature (101°C–26.7°C in 70 cm) was present in the sediments along the transect.

A high amount of sulfide was dissolved in the hot fluid from the bottom of HS6 (Table 2). Gas emitted from the bottom of HS6 predominantly contained CO_2_ (approximately 60% of total gas content) and a modest amount of H_2_ (approximately 0.3% of total gas content) was also detected. These anaerobic gasses were also detected from the bottom layer of the sediment at approximately 70°C (CO_2_: 40%, H_2_: 0.3%). These gases decreased with increasing distance from HS6. Oxygen was not detected in the gas emitted from the bottom of HS6, while hot fluid from the same point contained a modest amount of dissolved oxygen (1 ppm). Despite vigorous steam emission, the gas contents of the surface layer of HS6 were similar to those of atmospheric air. Dissolved sulfide also decreased with increasing distance from the bottom of HS6 (Table 2). This indicates the presence of an anaerobic zone near the bottom of HS6 and microaerobic to aerobic zones surrounding it. CO_2_, H_2_, and sulfide emitted from the bottom of HS6 could serve as a carbon source and as an electron donor for chemolithoautotrophic microbes in the anaerobic zone. Coarse-grained sandy sediments may allow diffusion of these anaerobic gasses and reducing compounds (sulfide) from the bottom of HS6 to the surrounding zones, while atmospheric air diffuses from the surface layer.

**Fig. 2:**
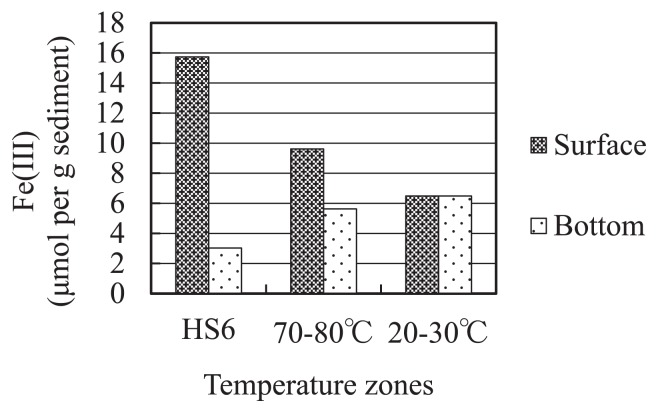
Fe(III) content of sediment samples from HS6 and its adjacent sites. Data are from a single trial.

Sandy sediments consist of volcanic rocks such as basalt, pyroxene, olivine, and pumice. These minerals are known to bear substantial amounts of iron and aluminum. The amount of Fe(III) in the surface sediments showed a clear declining trend with distance from the hot spot (15.7–6.5 μmol per g sediment), while those of bottom sediments showed an increasing trend (3.0–6.5 μmol per g sediment) (Fig. 2).

Thus, HS6 included physicochemical settings similar to those of a deep-sea hydrothermal vent, *i.e.* steep gradient of temperature, reducing power (H_2_ and sulfide) and CO_2_ emission from the subsurface, presence of anaerobic zone with high temperature, and the presence of abundant Fe(III). On the other hand, the steep gradient of oxygen discriminates HS6 from deep-sea hydrothermal vents, which is derived from anaerobic gas emission from the bottom layer and direct exposure to atmospheric air.

### Microbial community analyses

**Fig. 3:**
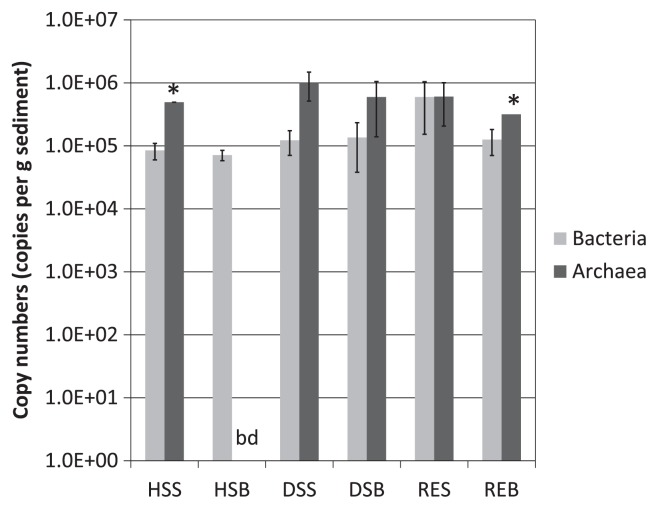
Comparison of bacterial and archaeal 16 S rRNA genes copy numbers. *data which were not detected in one sample of triplicate experiments. bd, below detection limit of 1.7×10^3^ copies. Error bars showing standard deviation.

**Fig. 4:**
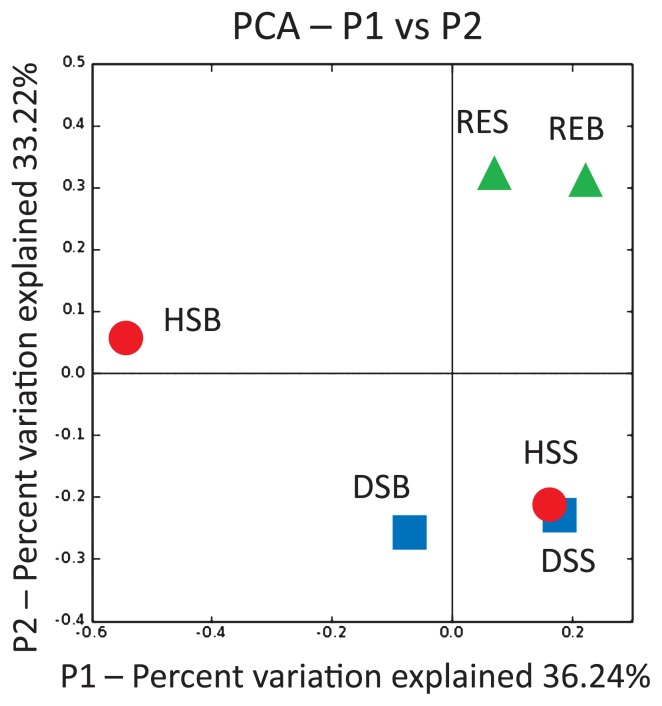
Communities clustered using normalized weighted-UniFrac PCA for bacterial communities. Each point represents an individual sample.

From the 12 clone libraries, 443 sequences with an average length of 597 bp (341–720 bp and 453–767 bp for *Archaea* and *Bacteria*, respectively) were investigated. Rarefaction analyses showed that bacterial communities are much more diverse than archaeal communities (Fig. S1). Normalized weighted UniFrac PCA revealed that both archaeal and bacterial communities of the HSB sample were distinct from those of other samples (Figs. S2 and 4, respectively). Bacterial community compositions of the samples clustered in three groups: high temperature and anaerobic sample (HSB), microaerobic to aerobic, high temperature samples (HSS, DSS, and DSB), and ordinary temperature samples (RES and REB) (Fig. 4). Quantitative PCR revealed the low biomass (7.1×10^4^–1.2×10^6^ 16S rRNA gene copies per g sediment for total bacteria and archaea) of this environment (Fig. 3).

**Fig. 5:**
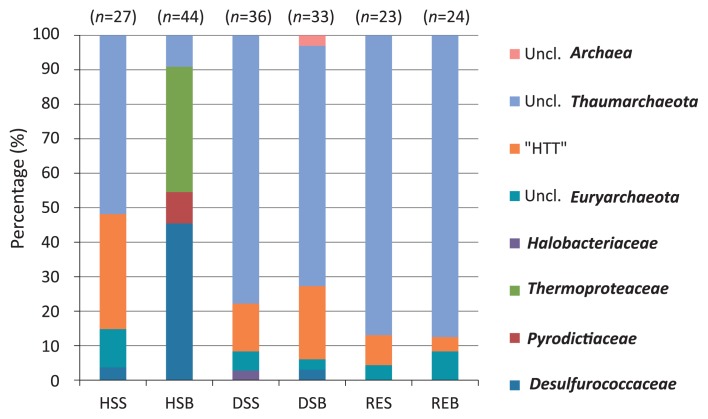
Comparison of taxonomic variation in the archaeal communities of the Yamagawa coastal hydrothermal field. Relative abundance of archaeal families observed in each sample are shown. Numbers in parentheses indicate the numbers of clone sequences. “HTT” (hydrothermal field *Thaumarchaeota*) indicates a cluster affiliated with sequences of *Thaumarchaeota* obtained from hydrothermal fields. Uncl., unclassified.

In archaeal communities, the most striking difference between HSB and other samples was the predominance of hyperthermophilic *Crenarchaeota* (*Desulfurococcaceae*, *Pyrodictiaceae*, and *Thermoproteaceae*) at HSB (Fig. 5). Sequences assigned to the family *Desulfurococcaceae* were closely related to the genus *Aeropyrum* (Table S1). In addition to the detection of relative clone sequences, the isolation of strains affiliated in this genus (Tanaka *et al.* unpublished data) and two novel strains of virus infecting the members of this genus (38, 39) ensured the inhabitation of this hyperthermophilic archaeon in this environment. The genus *Aeropyrum* consists of two species, *A. pernix* and *A. camini*, originally isolated from a shallow hydrothermal vent (53) and from a deep-sea hydrothermal vent (42), respectively. Both species are strictly aerobic heterotrophic hyperthermophiles. Clones assigned to the family *Pyrodictiaceae* were most closely related to *Geogemma indica* strain 296^T^ or clone sequences obtained from deep-sea hydrothermal vents (Table S1). The family *Pyrodictiaceae* consists of anaerobic hyperthermophiles optimally grow at temperatures above 100°C, such as the genera *Pyrodictium* (45, 58), *Geogemma* (22), *Hyperthermus* (70, 71), and *Pyrolobus* (5). The sequences assigned to the family *Thermoproteaceae* were related to the genus *Pyrobaculum* and most closely related to an isolate from Obama Hot Spring (Table S1), which is a coastal hot spring located approximately 180 km north of the Yamagawa coastal hydrothermal field. The members of the genus *Pyrobaculum* are hyperthermophilic facultative anaerobes. The members of the family *Pyrodictiaceae* and the genus *Pyrobaculum* grow heterotrophically or chemolithoautotrophically using molecular H_2_ as an electron donor.

**Fig. 6:**
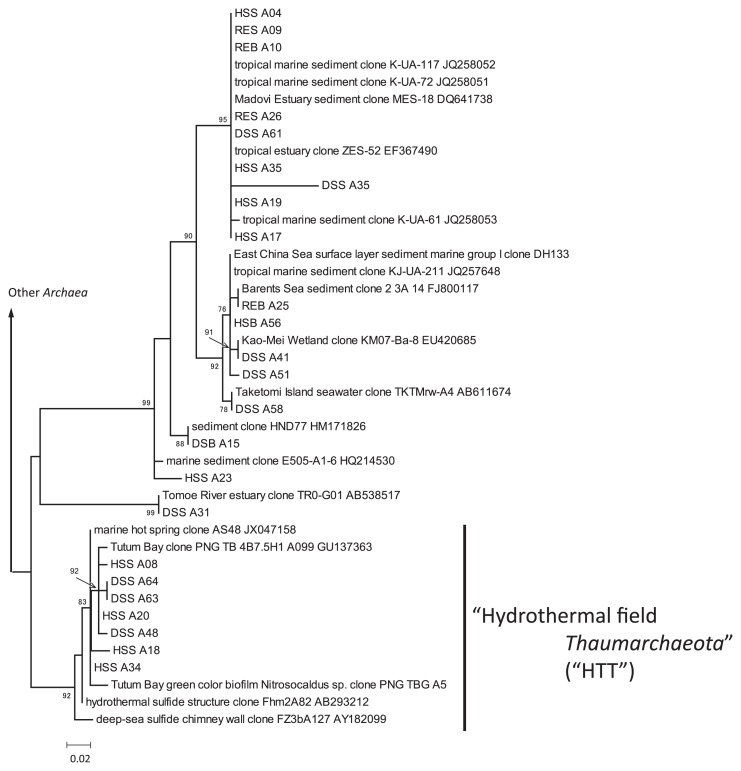
ML tree of the archaeal phylum *Thaumarchaeota*. ML bootstrap values ≥75% are shown at each node. Environmental clone sequences are described by their specific names and GenBank/EMBL/DDBJ accession numbers.

Archaeal sequences obtained from the samples other than HSB were dominated by sequences affiliated to *Thaumarchaeota*, a recently described archaeal phylum ubiquitously detected from various environments (7, 44). The archaeal ML tree indicated that the sequences affiliated to *Thaumarchaeota* were clustered in two groups (Fig. 6). One of the two (referred to as “HTT” group) consists of sequences obtained from hydrothermal fields around the world, such as Tutum Bay, an iron- and arsenic-rich shallow-sea hydrothermal field in Papua New Guinea (1, 37). Although, unfortunately, metal compositions of other environments are not available, it can be assumed that the hydrothermal sulfide structure and deep-sea chimney wall consist of a modest amount of iron, because the accumulation of iron by the mixing of hydrothermal fluids and cold ambient seawaters is the most important process in the formation of these metallic-sulfide structures. Despite its ubiquitous distribution, the phylum consists of few isolates (29, 63). Thus, the functions of the members of this phylum attracted our interest. In light of the distribution of “HTT” in HS6, members of this group might be adapted to iron-rich environments at either high or moderate temperatures, although considerable effort is needed to speculate the functions of microbes from their phylogeny.

**Fig. 7:**
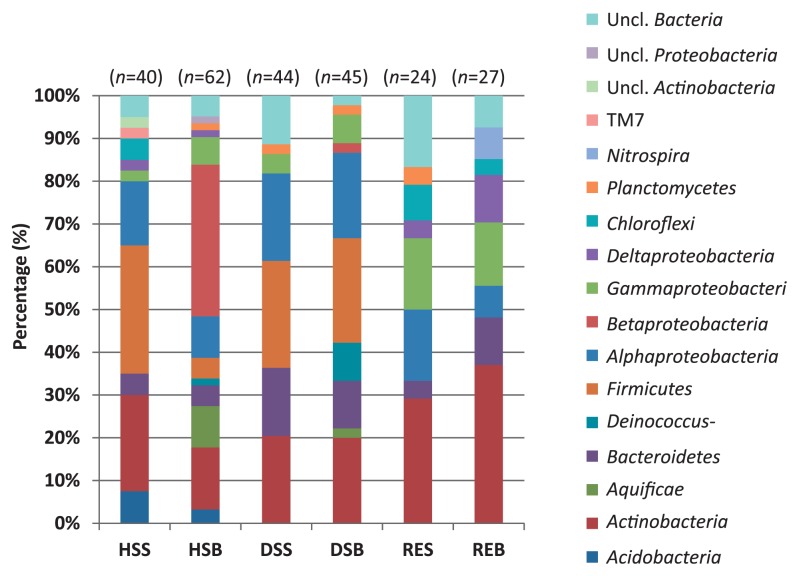
Comparison of taxonomic variation in the bacterial communities of the Yamagawa coastal hydrothermal field. Relative abundance of bacterial orders observed in each sample is shown. Numbers in parentheses indicate the numbers of clone sequences. Uncl., unclassified.

Overall, the bacterial community of the Yamagawa coastal hydrothermal field consists of diverse taxa, including both thermophiles and mesophiles. The only bacterial order shared by all samples was *Rhodobacterales* of the phylum *Alphaproteobacteria* (Fig. 7, Table S2). The order is phenotypically, metabolically, and ecologically diverse (18). However, many of them are aquatic and require NaCl for growth. Including *Rhodobacterales*, most bacterial clones were relative to clone sequences retrieved from marine environments around the world.

Like the archaeal communities, HSB community significantly differed from those of other samples. The phylum *Betaproteobacteria* represented the largest fraction (37.1%) in HSB (Fig. 7, Table S2). The sequences affiliated to this phylum, except one sequence, were closely related to the genus *Herbaspirillum* (Table S2). The species of this genus mainly comprise diazotrophic bacteria with potential for endophytic and systemic colonization of a variety of plants (13, 14, 28, 52, 64). All of the validly described species are mesophilic heterotrophs, except one autotrophic species (13). Contrary to the mesophilic physiology of the known isolates, clone sequences affiliated to this genus are often detected around hydrothermal fields (25, 60). In some studies, the predominantly detected *Herbaspirillum* is conjectured to be a result of contamination through DNA extraction or cloning procedures (62). However, while these environments harbored a steep temperature gradient, it can be assumed that the members of this genus were detected in hydrothermal fields due to the presence of mesophilic (micro) habitats. In case of the Yamagawa hydrothermal field, mesophilic zones may temporally occur around HSB due to tidal activity, thus providing favorable habitats for *Herbaspirillum*. In addition to nitrogen-fixing heterotrophic growth, some isolates affiliated to this genus are reported to grow chemolithoautotrophically using H_2_ and CO_2_ as their energy and carbon sources (13, 37). Considering the low biomass in HSB revealed by quantitative PCR (Fig. 3), dominantly detected *Herbaspirillum* may be inferred to be one of the primary producers in HSB during high tide. Another mesophilic bacterial phylum, *Firmicutes*, was also detected from high temperature samples (HSS, HSB, DSS, and DSB) (4.8–30.0%) (Fig. 7, Table S2). Unlike the genus *Herbaspirillum*, this order mainly consists of spore-forming bacteria (54). Thus, the detection could be the result of amplification of the 16S rRNA gene of their spores.

Two thermophilic bacterial phyla, *Aquificae* and *Deinococcus-Thermus*, were detected from HSB and DSB (Fig. 7). Allfoursequencesidentifiedas*Deinococcus-Thermus* were relatives of an isolate from a deep-sea hydrothermal vent, presumably representing a novel species affiliated to the genus *Rhabdothermus* (Kawaichi *et al.* in preparation). Like other genera in the order *Thermales*, the only described species of the genus *Rhabdothermus*, *R. arcticus*, is a heterotrophic thermophile (57). *R. arcticus* requires NaCl for its growth, grows under microaerobic conditions, and is also capable of anaerobic respiration in the presence of either nitrate or elemental sulfur. The other thermophilic bacterial phylum, *Aquificae*, is a group of thermophilic autotrophic bacteria capable of oxidation of molecular H_2_, thiosulfate, and S^0^, and reduction of O_2_, S^0^, and nitrate (17, 43, 48). The members of this phylum have been found in hot springs and deep-sea hydrothermal vents as primary carbon fixers (49, 50). Combining these findings with the results of quantitative PCR targeting bacterial and archaeal 16S rRNA genes showing that archaeal 16S rRNA genes were below the detection limit in HSB, it is suggested that members of *Aquificae* are the main primary producers, contributing more than autotrophic and mixotrophic archaea.

### Chemo-synthetic ecosystem in HS6

Our data showed the emission of H_2_, CO_2_, and sulfide from the bottom of HS6, and also the presence of high iron content and steep gradients of temperature around the hotspot. The emission of H_2_, CO_2_, and sulfide might provide favorable habitats for anaerobic chemolithoautotrophic microbes, and abundant ferric iron might be an energetically favorable electron acceptor for some microbes in anaerobic environments due to its high redox potential. It can also be assumed that sulfide provided from hot fluids will be oxidized abiotically by oxygen in the air or in ambient seawater, and then provide S^0^ or thiosulfate, which can serve as electron donors or electron acceptors for chemolithoautotrophic microbes. In fact, clone analysis revealed the presence of chemolithoautotrophic thermophiles capable of oxidation of H_2_ and oxidation or reduction of sulfur compounds (the bacterial order *Aquificales* and the archaeal families *Thermoproteaceae* and *Pyrodictiaceae*) in the HSB.

These findings inferred the presence of a microbial ecosystem in HS6 based on the chemical energy source provided by HSB. Namely, chemolithoautotrophic thermophiles, mainly *Aquificae*, function as primary producers using H_2_ or sulfur compounds as their energy source and CO_2_ as their carbon source. The organic compounds synthesized by them support the growth of thermophilic heterotrophs, so-called “chemo-synthetic microbial ecosystems.” Of course, as many mesophilic microbes were detected from the samples, these microbes may possibly serve as part of the energy and carbon sources for this ecosystem.

Some strains of the archaeal genus *Pyrobaculum* and most members of the archaeal family *Pyrodictiaceae* grow by Fe(III) reduction and also by reduction of sulfur compounds such as S^0^ and thiosulfate (8, 16, 22, 23, 24, 65). Despite the presence of iron-bearing rocks, no well-known bacterial iron reducers, such as the members of the genera *Geobacter* and *Shewanella*, were detected in this study. In fact, the isolation of an iron reducer affiliated to the phylum *Chloroflexi*, which is the first report of this ability in the phylum, was achieved using sandy sediment from this environment (26). Since the ability to grow by dissimilatory Fe(III) reduction is species or even strain specific, further studies, especially based on culture-dependent approaches, are necessary to reveal the effect of the high amount of iron in this environment on the bacterial (and also archaeal) community.

## Supplementary Information



## Figures and Tables

**Table 1:** Temperature distribution along the transect

Depth (cm)	Distance from HS6 (cm)										

	−100	−70	−60	−50	0	40	50	60	70	100	200
0	24.4	29.2	92.7	101	101	101	90	75	26.7	24.1	24
10	24.7	50	101	101	101	101	101	101	101	38.1	32.3
20	24.7	100	101	101	101	101	101	101	101	75.4	58.5
30	24.9	101	101	101	101	101	101	101	101	99	90.1 (25)^a^
40	25.3	100.8 (35)^a^	101	101	101	101	101	101	101	100.5 (35)^a^	nd

*nd*, not determined.

a Numbers in parentheses indicate depth.

**Table 2:** Physicochemical characteristics of hot fluids and gasses along the transect

Distance from HS6 (cm)	Depth (cm)	Temp. (°C)	Hot fluids			Gas contents (%)				
	
			pH^a^	DO (ppm)^a^	H_2_S (ppm)^a^	H_2_	O_2_	N_2_	CH_4_	CO_2_
0	0–5	101.0	nd	nd	nd	bd	22.74	77.24	bd	bd
0	30	101.0	7.6	1	0.8-over	0.28	bd	27.31	bd	57.48
50	30	101.0	6.1	1	0.8-over	0.26	5.07	59.23	bd	40.46
100	30	99.0	nd	nd	nd	bd	23.02	75.23	bd	4.11
150	25	91.8	nd	nd	nd	bd	23.56	78.91	bd	bd
REB^b^	30	24.0	7.9	5	0.2	bd	12.23	89.42	bd	bd

nd, not determined; bd, below the detection limits.

a The pH, dissolved O_2_ concentration and dissolved H_2_S concentration at the reference site refers to those of air-saturated seawater.

b REB, Bottom layer of the ordinary temperature zone (see text).
