# Supplementary material for: Bacterial and Archaeal Diversity in an Iron-Rich Coastal Hydrothermal Field in Yamagawa, Kagoshima, Japan
Source: Microbes Environ. 2013 Nov 21;28(4):405–13. doi: 10.1264/jsme2.ME13048 (PMC4070711; doi:10.1264/jsme2.ME13048)
Supplement: Supplementary file 1 [file 28_405_s1.pdf]

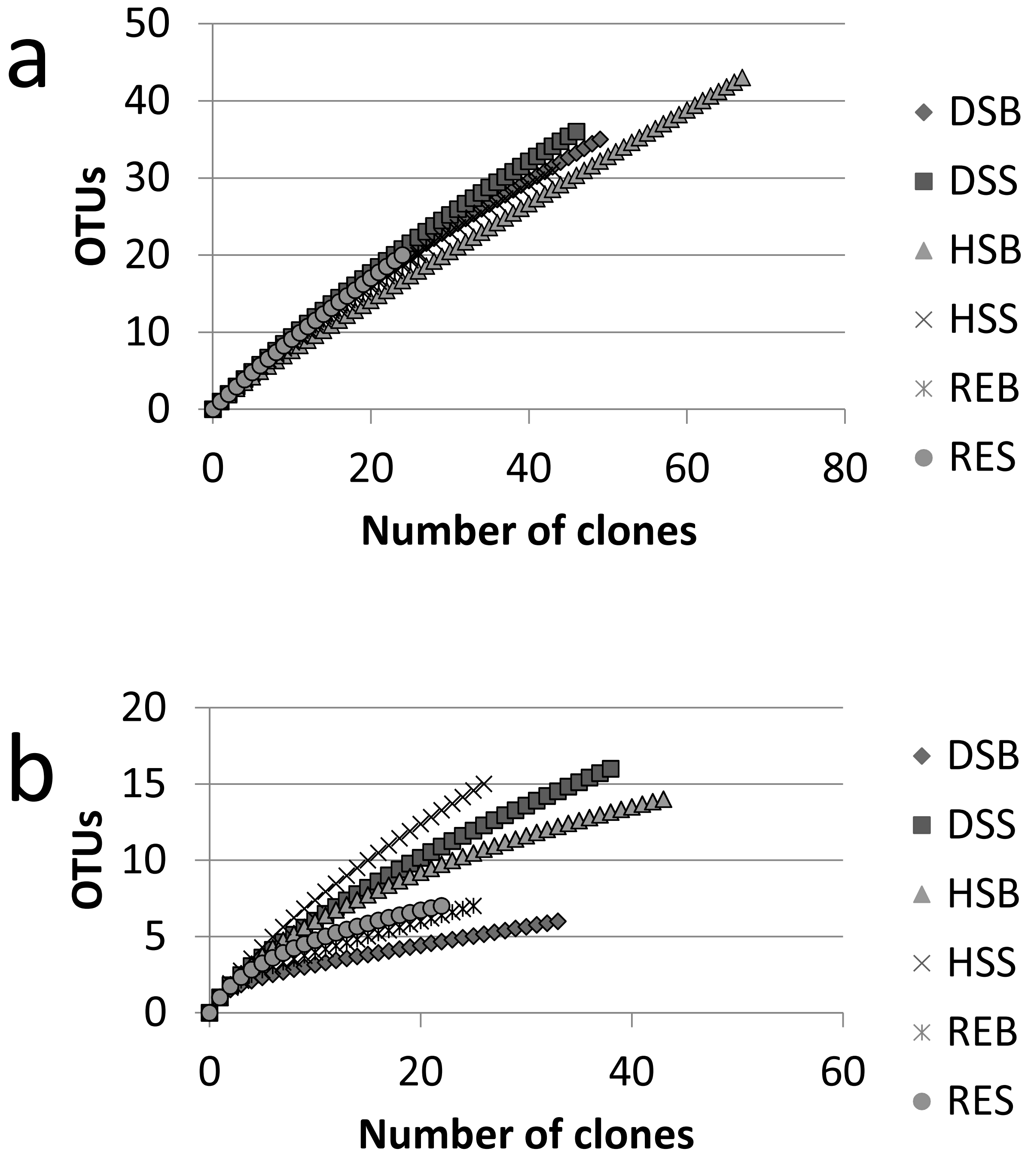

Fig. S1. Rarefaction curves for bacterial (a) and archaeal (b) clone libraries.

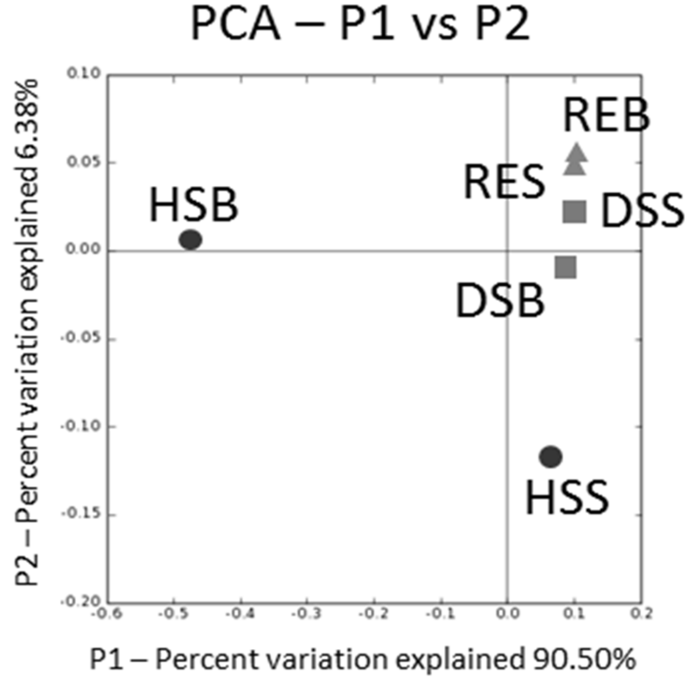

Fig. S2. Communities clustered using normalized weighted-UniFrac PCA for bacterial communities (a).
Each point represents an individual sample.
.

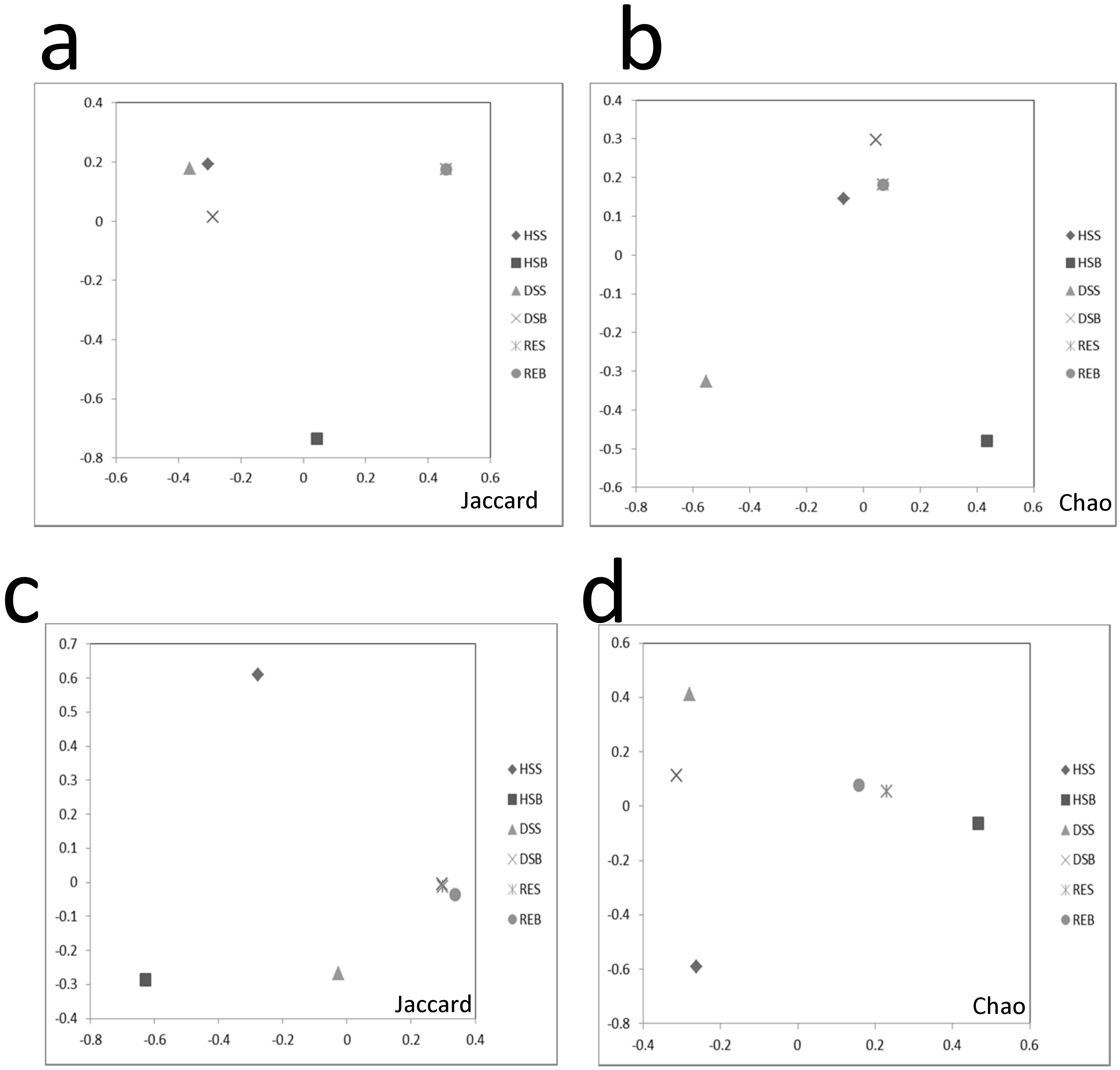

Fig. S3. Communities clustered using multidimensional scaling method for bacterial (a and b, Jaccard- and Chao-indices, respectively) and archaeal communities (c and d, Jaccard- and Chao-indices, respectively).
Each point represents an individual sample.
.

Table S1. Summary of archaeal 16S rRNA gene clone sequences identified from the Yamagawa coastal hydrothermal field.

Pylogenetic group	Representative clone	Number of clones						Closest match (NCBI BLAST)	Source	Accession no.	Similarity (%)
		HSS	HSB	DSS	DSB	RES	REB				
Crenarchaeota											
Desulfurococcaceae	DSB_A38				1			*Aeropyrum camini* strain SY1	deep-sea hydrothermal vent chimney	NR_040973	95
	HSB_A50		1					*Aeropyrum camini* strain SY1	deep-sea hydrothermal vent chimney	NR_040973	95
	HSB_A77		6					*Aeropyrum camini* strain SY1	deep-sea hydrothermal vent chimney	NR_040973	96
	HSS_A02	1	10					*Aeropyrum camini* strain SY1	deep-sea hydrothermal vent chimney	NR_040973	97
	HSB_A38		2					clone HTM1036Pn-A124	microbial mats on polychaete nests at the Hatoma Knoll	AB611454	99
	HSB_A68		1					clone HTM1036Pn-A124	microbial mats on polychaete nests at the Hatoma Knoll	AB611454	93
Pyrodictiaceae	HSB_A20		2					*Geogemma indica* strain 296	deep-sea hydrothermal vent sulfide chimney	DQ492260	98
	HSB_A46		1					clone TOTO-A1-15	Pacific Ocean, Mariana Volcanic Arc	AB167480	95
	HSB_A23		1					clone F99a113	nascent hydrothermal chimney	DQ228527	99
Thermoproteaceae	HSB_A18		3					*Pyrobaculum aerophilum* str. IM	boiling marine water hole at Maronti Beach	AE009441	98
	HSB_A21		4					*Pyrobaculum oguniense* TE7	Tsuetate Hot Spring, Kumamoto	CP003316	99
	HSB_A25		1					*Pyrobaculum* sp. M1T	Obama Hot Spring	AB302406	92
	HSB_A48		8					*Pyrobaculum* sp. M1T	Obama Hot Spring	AB302406	92
Euryarchaeota											
Halogranum	DSS_A40			1				clone Pa13	pustular mat from Hamelin Pool, Western Australia	DQ860873	96
Methanomicrobiales incertae sedis	RES_A17					1		33-FL31A00	mid-ocean ridge subseafloor	AF355880	93
Uncl. *Euryarchaeota*	DSB_A09				1			clone CWP-B5	seawater of western Pacific warm pool	HQ529816	99
	DSS_A42			1				clone TX4CA_37	alkaline-saline soil	EF690592	86
	DSS_A59			1				clone W50mCGB749	serpentinized dunite	JN002504	94
	HSS_A16	1						clone JSD14	arid desert sanddune	JQ071783	94
	HSS_A25	1						clone JSD14	arid desert sanddune	JQ071783	95
	HSS_A29	1						clone SAS_3D10	salt marsh sediment	FJ655628	88
	REB_A12						1	clone pYK04-19A-39	hydrothermal vent chimney structure in the Yonaguni Knoll IV	AB464800	88
	REB_A15						1	clone HKT-KArS14	Kutch desert soil	HE802746	96
Thaumarchaeota											
"HTT"	DSS_A48			1				clone PNG_TB_4B7.5H1_A099	Tutum Bay vent 4	GU137363	98
	HSS_A08	5		1	7	2	1	clone PNG_TB_4B7.5H1_A099	Tutum Bay vent 4	GU137363	99
	HSS_A20	1						clone PNG_TB_4B7.5H1_A099	Tutum Bay vent 4	GU137363	94
	DSS_A63			1				clone PNG_TBG_A52	green colored biofilm at Tutum Bay Vent 4	JN881612	90
	DSS_A64			2				clone PNG_TBG_A52	green colored biofilm at Tutum Bay Vent 4	JN881612	96
	HSS_A34	1						clone PNG_TBG_A52	green colored biofilm at Tutum Bay Vent 4	JN881612	94
	HSS_A18	2						clone AS48	Kalianda Island marine hot spring	JX047158	92
Other *Thaumarchaeota*	DSB_A15			2	1	2	1	clone HND77	Hainan island sediment	HM171826	99
	DSS_A31			1				clone TR0-G01	Tomoe River estuary	AB538517	99
	DSS_A35			1				clone K-UA-117	tropical marine sediment from Arabian Sea coast	JQ258052	92
	DSS_A41		1	1				clone KM07-Ba-8	Kao-Mei Wetland	EU420685	96
	DSS_A51					1		clone DH133A46	surface layer sediments from the East China Sea	JN590132	95
	DSS_A58	1						clone TKTMrw-A4	seawater outside a shallow submarine hot spring	AB611674	98
	DSS_A61			1				clone K-UA-72	tropical marine sediment from Arabian Sea coast	JQ258051	98
	HSS_A17	1						clone K-UA-72	tropical marine sediment from Arabian Sea coast	JQ258051	94
	HSS_A35	1						clone K-UA-72	tropical marine sediment from Arabian Sea coast	JQ258051	95
	HSB_A56	1	2	1				clone KJ-UA-211	tropical marine sediment from Arabian Sea coast	JQ257648	95
	HSS_A04	1						clone ZES-52	tropical estuary	EF367490	99
	REB_A10	6	1	20	20	13	17	clone ZES-52	tropical estuary	EF367490	99
	RES_A09			1	1	1		clone ZES-52	tropical estuary	EF367490	99

	HSS_A19	1						clone K-UA-61	tropical marine sediment from Arabian Sea coast	JQ258053	92
	HSS_A23	1						clone E505-A1-6	marine sediment from South China Sea	HQ214530	92
	REB_A25	1			1	2	3	clone 2 3A 14	sediment from Barents Sea	FJ800117	99
	RES_A26					1		clone MES-18	Madovi Estuary sediment	DQ641738	99
Uncl. *Archaea*	DSB_A04				1			clone Fhm5A01	hydrothermal sulfide structure	AB424704	99
Total number of clone		27	44	36	33	23	24				

Table S2. Summary of bacterial 16S rRNA gene clone sequences identified from the Yamagawa coastal hydrothermal field.

Pylogenetic group	Representative clone	Number of clones						Closest match (NCBI BLAST)	Source	Accession no.	Similarity (%)
		HSS	HSB	DSS	DSB	RES	REB				
Acidobacteria											
Gp4	HSS_B56	1						clone D20h16S176	rice paddy field soil	AB672214	97
Gp9	HSB_B70		1					clone SIMO-2326	Sapelo Island Microbial Observatory Dean Creek Marsh sampling site	AY711692	93
Gp21	HSB_B51		1					clone IBS1-001	Ibusuki coastal hot spring	AB703469	99
Gp22	HSS_B59	2						clone IBS1-142	Ibusuki coastal hot spring	AB703535	99
Actinobacteria											
Acidimicrobiales	HSB_B73		1					clone HG149	intestinal content and mucus from farmed seahorses	FN582327	93
	DSS_B46			1				clone 13c-79	ocean sediments	FJ626888	92
	HSS_B22	1						clone AKYG1106	farm soil adjacent to a silage storage bunker	AY921951	93
	REB_B43						1	clone Q10920	intertidal surface sediment	JX193415	96
Actinomycetales	HSB_B17		1					clone CVMbac10	petroleum deposit	JF922890	99
	HSB_B47		1					*Leucobacter aridicollis* strain L9	chromium contaminated environment	NR_042288	99
Euzebyales	HSB_B59		1					clone Si07 19	permeable coral reef sands	FR851699	98
Uncl. *Actinobacteria* (class)	DSB_B01				1			clone Ld1-14	North Yellow Sea sediments	GQ246409	93
	DSB_B03			1	2	1	1	clone Ld1-14	North Yellow Sea sediments	GQ246409	93
	DSS_B24			1	1			clone DH133B17	surface layer sediment of the East China Sea	JN672635	92
	DSS_B55			1				clone MS-K54	calcareous sandy sediment, Mallorca, Balearic Islands	FJ949206	96
	DSS_B57			1				clone Q31005	intertidal surface sediment	JX193432	92
	DSS_B17			1				clone RII-AN095	sediments from Rodas Beach polluted with crude oil	JQ580474	95
	REB_B22	1				1	1	clone RII-AN095	sediments from Rodas Beach polluted with crude oil	JQ580474	94
	HSB_B53		1					clone RII-OX022	sediments from Rodas Beach polluted with crude oil	JQ580109	92
	HSB_B80		1					clone FII-OX065	sediments from Figueiras Beach	JQ579714	92
	HSB_B56		1					clone DH133B17	surface layer sediment of the East China Sea	JN672635	95
	HSS_B16	1						clone DH133B17	surface layer sediment of the East China Sea	JN672635	89
	DSS_B12			1				clone Si06 11	permeable coral reef sands	FR851637	93
	DSS_B47			2				clone Si06 11	permeable coral reef sands	FR851637	94
	HSB_B65	3	1					clone Si06 11	permeable coral reef sands	FR851637	92
	HSS_B25	1						clone Si06 11	permeable coral reef sands	FR851637	94
	HSS_B50	1						clone Si06 11	permeable coral reef sands	FR851637	87
	REB_B27						5	clone Si06 11	permeable coral reef sands	FR851637	93
	REB_B29						1	clone 1112864242305b	loamy sand from a tomato planted field	HQ120429	92
	RES_B26					1		clone B4-1	soil	HQ693487	96
	DSB_B88				1			clone 20BSU31	poplar tree microcosm, bulk soil	AJ863192	85
	HSS_B44	2	1		4	4	1	clone 20BSU31	poplar tree microcosm, bulk soil	AJ863192	92
Aquificae											
Aquificales	HSB_B27		1					*Aquifex aeolicus* VF5		AE000657	98
	HSB_B63		1					*Aquifex aeolicus* VF5		AE000657	93
	HSB_B58		3		1			clone pMARB06_56	chimney rock fragments of Lucky strike deep sea hydrothermal vent field	AB496516	95
	HSB_B68		1					clone pMARB06_56	chimney rock fragments of Lucky strike deep sea hydrothermal vent field	AB496516	88
Bacteroidetes											
Flavobacteriales	DSS_B13			1				*Salinimicrobium* sp. BB-My20	tidal sediment	JF340052	96
	DSS_B21			1				clone MBFOS-06	oyster shell	EU369165	93
	DSS_B54			2				clone MBFOS-06	oyster shell	EU369165	96
Sphingobacteriales	DSB_B65				1			clone IBS2-111	Ibusuki coastal hot spring	AB703571	94
	DSB_B73		1		2			clone IBS2-111	Ibusuki coastal hot spring	AB703571	98
	HSB_B87		1					clone IBS2-111	Ibusuki coastal hot spring	AB703571	95
	DSB_B44				1			*Pontibacter* sp. HMC5104	solar saltern	FJ903180	93
	HSS_B61	1		1				clone ARTE12_243	coastal sediment	GU230422	90
	REB_B20			2	1		3	clone ARTE12_243	coastal sediment	GU230422	93
Uncl. *Bacteroidetes*	HSB_B67		1					clone SiDMar08M99	reverse osmosis membrane from a desalination plant	GU326759	94
	HSS_B13	1						clone ARTE12_243	coastal sediment	GU230422	89
	RES_B45					1		clone HKT937	activated biomass from a CETP after induction with salicylate	DQ989464	90
Chloroflexi											
Caldilineales	HSS_B58	1						clone IBS2-095	Ibusuki coastal hot spring	AB703568	97
Uncl. *Chloroflexi*	REB_B17	1				2	1	clone Er-LAYS-46	estuary sediment and soil slurry	GU180180	94

Deinococcus-Thermus											
Thermales	DSB_B21				1			Thermophilic bacterium IH55	Iheya North hydrothermal field deep-sea hydrothermal vent	AB465709	97
	DSB_B53				1			Thermophilic bacterium IH55	Iheya North hydrothermal field deep-sea hydrothermal vent	AB465709	91
	DSB_B59				1			Thermophilic bacterium IH55	Iheya North hydrothermal field deep-sea hydrothermal vent	AB465709	93
	HSB_B91		1					Thermophilic bacterium IH55	Iheya North hydrothermal field deep-sea hydrothermal vent	AB465709	90
	DSB_B79				1			Thermophilic bacterium IH55	Iheya North hydrothermal field deep-sea hydrothermal vent	AB465709	97
Firmicutes											
Bacillales	DSB_B12				1			*Bacillus* sp. 142213	mangrove soil	EF522812	99
	HSB_B10		1					*Bacillus* sp. 'Mali 10'	African desert dust air sample	AY211104	99
	HSB_B23		2					clone ET_B_2b07	biofilm of extubated endotracheal tube of ICU patient	FJ557411	99
	DSB_B52	3		2	4			*Paenisporosarcina* sp. NSP480	North Sea Sponge	FR750928	92
	DSB_B71				1			*Paenisporosarcina* sp. NSP480	North Sea Sponge	FR750928	91
	DSS_B53			1				*Paenisporosarcina* sp. NSP480	North Sea Sponge	FR750928	93
	DSS_B26			1				*Sporosarcina* sp. K2-28-011	deep-sea sediment core from Okinawa Trough, Pacific Ocean	DQ356965	91
	DSS_B38	6		5	4			*Sporosarcina* sp. K2-28-011	deep-sea sediment core from Okinawa Trough, Pacific Ocean	DQ356965	97
	HSS_B49	1						*Sporosarcina* sp. K2-28-011	deep-sea sediment core from Okinawa Trough, Pacific Ocean	DQ356965	95
	DSS_B59			1				*Sporosarcina* sp. K2-4-037	deep-sea sediment core from Okinawa Trough, Pacific Ocean	DQ356970	92
	HSS_B33	1		1	1			*Sporosarcina* sp. K2-4-037	deep-sea sediment core from Okinawa Trough, Pacific Ocean	DQ356970	94
	HSS_B23	1						clone HY352	paddy soil enrichment culture for degrading DDT	JX473608	96
Nitrospira											
Nitrospirales	REB_B16						1	clone wb1_F07	Nullarbor caves, Australia	AF317764	96
	REB_B45						1	clone 9M4I_008	East Pacific Rise	JQ287168	98
Planctomycetes											
Planctomycetales	DSB_B67				1			clone SW2-7-8F	Red Sea water	FJ895223	91
	HSB_B82		1					clone BD72BR161	marine sediment from the South China Sea	GU363004	95
	RES_B06					1		clone FII-TR008	sediments from Figueiras Beach	JQ579803	98
	DSS_B14			1				clone RII-OX010	sediments from Rodas Beach polluted with crude oil	JQ580097	92
Alphaproteobacteria											
Rhodobacterales	HSS_B21	1						clone Cobs2TisF11	Calcinus obscurus abdominal flora	EU246824	97
	DSB_B66				1			*Loktanella* sp. HME8261	sea water	JQ687344	92
	DSB_B75				1			*Loktanella* sp. HME8261	sea water	JQ687344	93
	DSB_B48				3			*Loktanella* sp. HME8261	sea water	JQ687344	99
	DSB_B77						1	*Loktanella* sp. HME8261	sea water	JQ687344	91
	DSS_B51			2				clone HTM1039S-B7	chimney structure at hydrothermal active area of Hatoma Knoll	AB611120	97
	DSS_B25			1				clone 2S-5m-6	South China Sea	GU062032	96
	DSS_B27			1				clone OO.P3.LT.67.ab1	coastal water	HQ821747	96
	DSB_B63				1			clone Bac92_Flocs	floating microbial flocs in brackish water of a shrimp hatchery pond	AB491852	90
	DSB_B74				1			clone Tat-08-009_43_28	Tower Geyser, El Tatio Geyser Field dry surface microbial mats	GU437462	89
	HSS_B47	1						clone Tat-08-009_43_28	Tower Geyser, El Tatio Geyser Field dry surface microbial mats	GU437462	93
	HSS_B54	1						clone Tat-08-009_43_28	Tower Geyser, El Tatio Geyser Field dry surface microbial mats	GU437462	94
	DSS_B58			1				clone J20-11	surface seawater	HQ166775	95
	HSB_B61		1					clone A51	natural biofilm with Ag NP treatment	GU066483	94
	HSB_B69		1					clone BM89MF1BB1	coral-associated	AF365447	96
	HSB_B88		1					clone Z-1-199	ballast water	FJ666157	94
	REB_B25						1	clone BJGMM-1s-235	Yellow River Delta soil	JQ800758	92
	RES_B21				1	1		clone 409_G08_F	Rainbow-2 deep-sea hydrothermal vent deposit at Mid-Atlantic Ridge	HQ894291	96
	RES_B43					1		clone CanalPalD39	highly copper-contaminated marine sediment	JX162134	93
	HSS_B26	1						clone RESET_213E09	East Pacific Rise	JN874343	96
Sphingomonadales	DSB_B47				1			clone RESET_213A09	East Pacific Rise	JN874381	95
	DSS_B18			1				clone RESET_213A09	East Pacific Rise	JN874381	98
	DSS_B28			1				Bacterium SCS_30Z0_1	South China Sea slope sediment	HM598288	95
	DSS_B30	1		1				*Erythrobacter aquimaris* strain HM-1-80	sponge	JX463484	98
	HSB_B22		1					clone LIB013_009_D01	drinking water biofilm	JX084343	99
Rhizobiales	HSB_B44		1					clone H5Ba35	oxic rice field soil	AY360587	99
Caulobacterales	HSS_B39	1						clone 9	marine sediment	FJ797345	94
Rhodospirillales	HSB_B62		1					clone p763_b_4.31	hydrothermal sediments from Okinawa Trough, Yonaguni Knoll IV	AB305502	92
Rhizobiales	DSS_B15			1				clone W04MLC05	sponge tissue	EF630069	94
Uncl. *Alphaproteobacteria*	RES_B01					1		clone 20MH2_PL5	Mussimilia hispida healthy coral MH2	GU199944	98
	RES_B02					1		clone TX2_4B18	extreme saline-alkaline soil of the former lake Texcoco	JN178173	92
Betaproteobacteria											

Burkholderiales	HSB_B01		22		1			*Herbaspirillum* sp. clone A4H6M9	Nilaparvata lugens midgut	GQ206314	99
Gammaproteobacteria											
Alteromonadales	DSS_B37			1				*Marinobacter sediminum* strain R65		NR_029028	95
Xanthomonadales	HSB_B55		1					*Stenotrophomonas* sp. 2345	maize	JX174222	99
	HSB_B20		1					*Lysobacter* sp. YIM 77875	geothermal soil	JQ746036	99
Uncl. *Gammaproteobacteria*	DSB_B16				1			clone IBS2-006	Ibusuki coastal hot spring	AB703549	99
	DSB_B22				1			clone NdSurf190	surface sediment, Canal Saint Antoine, Gulf of Fos	FJ753238	92
	DSB_B57				1			clone L3B11-2	coral mucus	FJ930590	98
	DSS_B01			1				clone TI-42	Tedania ignis (sponge) associated	GU981979	96
	HSB_B49		1					clone 6mMLC04R	sponge tissue	EF630126	94
	HSB_B95		1					clone FeOrig_B_120	Eel River Basin methane seep sediment	GQ357028	93
	HSS_B35	1						clone BATS10_177	Epibiont community attached to *Trichodesmium* colony	GU725997	88
	REB_B12						1	clone L3B11-2	coral mucus	FJ930590	91
	REB_B19						1	clone KspoA2	Desmacidon sponge from deep-sea coral reef off Norway	EU035919	94
	REB_B38						1	clone SH100_100m_clone_6	marine	HQ173722	97
	REB_B46					1	1	clone B185	deep sea sediment from the western Pacific Warm Pool	AY375060	97
	RES_B24					1		clone CaletaPalS74	highly copper-contaminated marine sediment	JX162155	90
	RES_B28					1		clone F9P122000_S_N13	Northeast subarctic Pacific Ocean, 2000 m depth	HQ673975	96
	RES_B40					1		clone A5-054	Jiaozhao Bay sediment	JN977277	98
Deltaproteobacteria											
Myxococcales	HSS_B19	1						clone AO56	marine reef sandy sediment	FJ358876	89
Uncl. *Deltaproteobacteria*	HSB_B64		1					clone 16B_232	Lagoon sediment	AM501756	91
	REB_B30						1	clone F.c-10	shrimp intestine	GU225840	85
	REB_B31						1	clone HM101	rhizosphere soil from rice field	AM909844	95
	REB_B33						1	clone KT0_G5	marine surface sediment	JN621467	91
	RES_B37					1		clone CMOC10	pilot-scale bioremediation process of a hydrocarbon-contaminated soil	AM935994	95
Uncl. *Proteobacteria*	HSB_B71		1					clone FII-TR143	sediments from Figueiras Beach	JQ579938	86
TM7 candidate division											
TM7 genera incertae sedis	HSS_B52	1						clone ULoh_187	hydrothermal vent mat from Upper Lohiau vent	JF320765	93
Uncl. *Bacteria*	REB_B05						1	clone SIMO-731	Sapelo Island Microbial Observatory Dean Creek Marsh sampling site	AY712268	92
	HSS_B32	1						clone IBS1-001	Ibusuki coastal hot spring	AB703469	96
	HSS_B46	1						clone ALVIN28a_8B_FF	sediment	FJ197530	95
	HSB_B66		1					clone 06-03-57	surface biofilm in estuarine seawater	DQ153138	86
	RES_B25					1		clone ELB16-214	lake water, East Lobe, Antarctica	DQ015814	93
	DSS_B44			2				clone FL0428B_PF10	Frasassi cave system, anoxic lake water	FJ716411	91
	DSB_B69				1			clone MPB1-182	aquatic moss pillars from Hotoke-Ike lake, Antarctica	AB630564	84
	RES_B04					1		clone 3G1820-56	Gulf of Mexico sediment	DQ431899	96
	HSB_B81		1					clone MidBa13	Pearl River Estuary sediments at 22 cm depth	EF999369	92
	DSS_B29			1				clone XF1E11	*Xestospongia muta* (marine sponge)	HQ270413	89
	RES_B08					1		clone PNG_TBG_B104	Tutum Bay vent 4	JN881637	93
	DSS_B41			1				clone Creta1-H09	oxic surface sediments of eastern Mediterranean Sea	AY534079	88
	DSS_B45			1				clone BJGMM-1s-302	Yellow River Delta soil	JQ800788	95
	REB_B40						1	clone 6mMLE08R	sponge tissue	EF630170	90
	RES_B48					1		clone KMS200711-033	maize-sweet potato cropland soil	EU881300	91
	HSB_B93		1					clone ATTNROCT093	coral	GU184693	94
Total number of clone		40	62	44	45	24	27				
